# Using Social Media to Improve Mental and Physical Health Literacy: The Meeting of Arts and Sciences

**DOI:** 10.1192/bjo.2022.143

**Published:** 2022-06-20

**Authors:** Lopez Okhiai, Jiann Lin Loo

**Affiliations:** Wrexham Maelor Hospital, Wrexham, United Kingdom

## Abstract

**Aims:**

Generation Z and millennials are tech-savvy and they learn more from videos compared to books. On average young people from the digital age spend more than five hours on digital gadgets. Innovative use of social media technology will improve the access to health information amongst this group of users. This article aims to share the project of using short video clips in social media, combined with poetry to improve mental and physical health literacy.

**Methods:**

Short video clips (ranging from one to three minutes) were produced out of passion by the first author using the elements of poetry, rhyming, humour, artistic expressions, simulated play of clinical scenarios and news reporting style which depends on the creativity and suitability of the content. The production process includes initial conceptualisation, script drafting and editing, video-recording using a smartphone, and subsequent editing using phone and Canva software. Subtitles and captions were added to increase accessibility. The videos were uploaded in Instagram, Twitter, and TikTok under the name of “dr_lokai”. There is no external funding involved. The cost involved included subscription of editing software and the purchase of recording equipment.

**Results:**

The project was first conceptualised in 2014. Total videos produced so far is 70. The topics of mental health included both normal psychological topics (mental health, self-reflective practice, self-motivation, self-compassions, and self-actualisation) and disorder-related topics (delirium, generalised anxiety disorder, emotionally-unstable personality disorder, attention-deficit hyperactivity disorder); while the physical health topics included cardiology, dermatology, infectious diseases, etc.). There were also videos on stigma, interesting contemporary topics around public health and healthcare education. One of the videos was a collaborative work with The Royal College of Physicians, elaborating on the personal and non-clinical facet of journey in medical school. As of the day of submission, the number of followers was 1710. Qualitative feedback from the audiences was generally positive. There were frequent requests from audiences for videos on specific medical topics.

**Conclusion:**

A creative generation requires a creative approach in outreach. The strength of this initiative is the low-cost production nature and it is freely accessible by anyone with internet access. In the future, more videos which involve debunking medical myths and history of medicine can be added. The main challenge is finding time to write the script, rehearse and record. Although the effectiveness and efficiency of this innovative initiative requires a systematic evaluation, passions in sharing medical knowledge using social media have kept this initiative alive.

